# Projection datasets of city- and grid-level building energy consumption for Hubei Province, China

**DOI:** 10.1016/j.dib.2019.104952

**Published:** 2019-12-05

**Authors:** Han Chen, Wenying Chen

**Affiliations:** Institute of Energy, Environment and Economy, Tsinghua University, Beijing 100084, China

**Keywords:** Building sector, Energy consumption, Emission reduction, City

## Abstract

This data article takes a typical low-carbon pilot province in Middle China (Hubei) as an example to present the pathway of building energy conservation and emission reductions for different cities. The data contains middle-to-long-term predictions of provincial socioeconomic factors (Gross Regional Production, population and urbanization rate), based on which building sector energy consumption under the base scenario could be estimated on provincial scale. Besides, energy demand and structures of the building sectors in cities from various categories are also provided by considering the spatial heterogeneity of city-level economic development and energy use intensities. This dataset could be used to calculate building sector emission reduction potentials on city scale so as to fill in the research gap of mitigation pathway modeling for multiple cities. Moreover, it also proposes a reasonable and convenient approach to allocate provincial targets concerning emission intensity and total amount control. Finally, the data offers high-resolution gridded projections for building energy consumption, which could be expanded to other sectors and cities to assist in more refined urban governance and atmospheric and climate modeling. The data presented herein are associated with the research article “Carbon mitigation of China's building sector on city-level: pathway and policy implications by a low-carbon province case study” [1].

**Table:** Specifications Table

Subject area	Energy
More specific subject area	Renewable Energy, Sustainability and the Environment
Type of data	Table, figure, text file
How data was acquired	Data were acquired from the provincial/city statistics, government development plans and a building energy downscaling model.
Data format	Analyzed and raster in ASCII form
Experimental factors	The raw data collected were organized in spreadsheets and then be calibrated to base-year data. Cities were grouped into four classes according to their emission intensity and economic development.
Experimental features	Spatial downscaling and scenario comparisons
Data source location	Hubei Province, China.
Data accessibility	Data are provided in the article
Related research article	Chen, H., Chen, W., 2019. Carbon mitigation of China's building sector on city-level: pathway and policy implications by a low-carbon province case study. J. Clean. Prod. 224, 207–217.

**Table:** 

**Value of the Data** • The dataset includes socioeconomic and technology related parameters required by modeling building energy consumption for cities at different development stages. • The dataset could shed light on the pathway for the building sector of other low-carbon pilot regions to achieve early emission peaking. • The dataset could help decompose provincial targets about building energy transition to cities. • Gridded energy consumption dataset could generate high-resolution emission projections to meet the data demand of air quality and climate models. • High-resolution building energy use datasets could be further combined with visualization platform to improve urban governance efficiency

## 1 Data

The data consist of key parameters and assumptions required by modeling future evolutions of city- and grid-level building energy consumption [1]. Table 1 listed province-level building energy consumption of three sub-sectors by fuel type, in which data of 2015 is collected from energy statistical yearbook and the values of 2020–2030 are predicted by the combination of trend analysis concerning total energy consumption and its structure. To be more specific, total amount is specified by provincial mid-term energy development and emission control targets, while the demand increase rate of individual energy type is forecasted based on temporal extrapolation of historical records. When it comes to city-level carbon emission projections under different scenarios (Table 2), the relationship between historical socioeconomic data (Supplementary Material Table 1) and energy use intensity is drawn first, then future trajectory is estimated according to the characteristics of base and policy scenarios (Supplementary Material Table 2). Energy use intensities under S1 (base scenario), are assumed to be driven only by socioeconomic factors. And policy scenario S2 and S3 take into account electricity and renewable energy share increases and higher efficiency of building energy technologies, respectively. Parameters regarding clean energy proportions and efficiency improvement of the province were set based on literature review [[2], [3], [4], [5]]. Then these parameters were adjusted for different groups of cities according to their socioeconomic development status. Additionally, natural gas consumption was used as an example to display grid-level energy data (ASCII file in the Supplementary material), which was generated by spatial downscaling from city-level calculations.

**Table 1:** Evolution of building energy consumption of Hubei Province during 2015–2030 modeled under S1 (10^4^ tce).

		Coal	Oil	Natural Gas	Electricity
Urban	2015	154.88	66.91	82.13	226.45
	2020	147.79	71.18	155.17	292.98
	2025	114.78	95.11	253.21	457.88
	2030	88.01	97.69	341.75	589.15
Rural	2015	255.24	70.020	5.22	116.39
	2020	304.82	81.86	9.70	134.28
	2025	286.95	116.24	15.83	173.34
	2030	242.02	126.42	21.36	200.31
Commercial	2015	350.52	75.04	85.90	248.38
	2020	351.01	85.42	166.81	292.98
	2025	286.95	110.96	300.69	425.18
	2030	234.69	109.18	427.19	589.15

**Table 2:** Direct carbon emissions (Mt CO_2_) from the building sector of 17 prefectural cities in Hubei Province under different scenarios.

	S1				S2				S3			
	2015	2020	2025	2030	2015	2020	2025	2030	2015	2020	2025	2030
WH	6.62	8.18	9.66	10.57	6.62	7.21	7.20	7.07	6.62	6.49	6.12	5.65
HS	1.09	1.20	1.31	1.31	1.09	1.16	1.23	1.20	1.09	1.11	1.11	1.01
SY	1.47	1.62	1.73	1.66	1.47	1.58	1.63	1.54	1.47	1.50	1.47	1.30
YC	2.05	2.52	2.85	3.08	2.05	2.34	2.42	2.45	2.05	2.18	2.12	2.03
XY	2.57	3.09	3.50	3.65	2.57	2.88	2.98	2.91	2.57	2.68	2.61	2.41
EZ	0.56	0.55	0.61	0.61	0.56	0.52	0.53	0.50	0.56	0.48	0.47	0.42
JM	1.25	1.38	1.51	1.55	1.25	1.34	1.41	1.41	1.25	1.27	1.26	1.19
XG	2.14	2.29	2.36	2.23	2.14	2.22	2.21	2.04	2.14	2.11	1.99	1.72
JZ	2.45	2.58	2.61	2.42	2.45	2.55	2.53	2.31	2.45	2.47	2.32	2.00
HG	2.87	3.31	3.31	2.98	2.87	3.27	3.22	2.87	2.87	3.17	2.95	2.47
XN	1.05	1.18	1.26	1.23	1.05	1.15	1.18	1.12	1.05	1.09	1.06	0.94
SZ	0.99	1.10	1.18	1.13	0.99	1.08	1.15	1.09	0.99	1.05	1.05	0.94
ES	1.73	1.98	1.97	1.74	1.73	1.93	1.87	1.61	1.73	1.83	1.68	1.36
XT	0.58	0.65	0.74	0.75	0.58	0.64	0.71	0.72	0.58	0.62	0.65	0.62
QJ	0.54	0.60	0.65	0.65	0.54	0.59	0.60	0.59	0.54	0.56	0.54	0.50
TM	0.40	0.45	0.49	0.49	0.40	0.45	0.47	0.46	0.40	0.43	0.43	0.40
SNJ	0.03	0.04	0.04	0.04	0.03	0.04	0.04	0.03	0.03	0.04	0.03	0.03

Note:

WH: Wuhan; HS: Huangshi; SY: Shiyan; YC: Yichang; XY: Xiangyang; EZ: E'zhou; JM: Jinmen; XG: Xiaogan; JZ: Jinzhou.

HG: Huanggang; XN: Xianning; SZ: Suizhou; ES: Enshi; XT: Xiantao; QJ: Qianjiang; TM: Tianmen; SNJ: Shennongjia.

## 2 Experimental design, materials, and methods

**Fig. 1:**
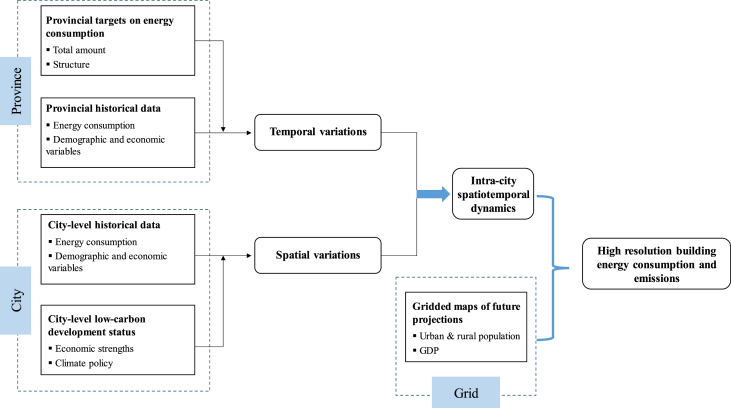
General workflow chart of data preparation.

The data presented in this article aim to provide future building energy consumption and emissions with higher resolution, which could be applied to climate modeling and more refined low-carbon management. The dataset incorporates information on three geographic/administrative levels and is produced by using the systematic downscaling framework described in Ref. [6]. First, provincial statistics [7] were employed to identify the historical trends of total energy demand and the share of the building sector in it. Then city-level energy statistics such as household and commercial natural gas and electricity use were used to describe the distribution patterns of building energy use intensity by quantitatively linking them to socioeconomic parameters. These trend analyses derived from past data could then be used to model the spatial and temporal variations of future building energy demand for the province. More specifically, grid-level urban/rural population and Gross Domestic Production (GDP) were incorporated into these relationships to generate spatial proxies for energy consumption distributions within cities. The above process could be generalized as shown in Fig. 1.

## References

[1] Chen H., Chen W.Y. (2019). Modelling building's decarbonization with application of China TIMES model. J. Clean. Prod..

[2] IEA (2015). Building Energy Use in China: Transforming Construction and Influencing Consumption to 2050. IEA Publication in Collaboration with Tsinghua University Building Energy Research Center (BERC). https://www.iea.org/publications/freepublications/publication/PARTNERCOUNTRYSERIESBuildingEnergy_WEB_FINAL.pdf.

[3] Shi J.C., Chen W.Y., Yin X. (2016). Modelling building's decarbonization with application of China TIMES model. Appl. Energy.

[4] Zhou N., Fridley D., McNeil M., Zheng N., Ke J., Levine M. China's Energy and Carbon Emissions Outlook to 2050. Reports from Lawrence Berkeley National Laboratory (LBNL). https://china.lbl.gov/sites/all/files/lbl-4472e-energy-2050april-2011.pdf.

[5] Yu S., Eom J., Evans M., Clarke L. (2014). A long-term, integrated impact assessment of alternative building energy code scenarios in China. Energy Policy.

[6] Chen H., Chen W.Y. (2019). Potential impact of shifting coal to gas and electricity for building sectors in 28 major northern cities of China. Appl. Energy.

[7] Hubei Statistics Bureau. Hubei Statistical Yearbook 2001-2018. China Statistics Press, Beijing.

