# Supplementary material for: Projection datasets of city- and grid-level building energy consumption for Hubei Province, China
Source: Data Brief. 2019 Dec 5;28:104952. doi: 10.1016/j.dib.2019.104952 (PMC6921097; doi:10.1016/j.dib.2019.104952)
Supplement: Multimedia component 1 [file mmc1.docx]

**Supplementary Material**

Table 1 Assumptions of macroeconomic drivers for Hubei Province under all scenarios

|  | Total population (ten thousand) | GDP (billion RMB) | Urbanization rate |
| --- | --- | --- | --- |
| 2015 | 5851.5 | 2955.1 | 0.57 |
| 2020 | 6100.3 | 4440.0 | 0.62 |
| 2025 | 6180.8 | 6315.2 | 0.67 |
| 2030 | 6088.5 | 8571.3 | 0.70 |

Table 2 Assumptions of key parameters for different groups of cities under different scenarios

|  | City type | S1 | | | |
| --- | --- | --- | --- | --- | --- |
|  |  | 2015 | 2020 | 2025 | 2030 |
| Share of electricity and renewable energy (%) | A | 43 | 46 | 48 | 50 |
|  | B | 41 | 42 | 46 | 49 |
|  | C | 26 | 28 | 32 | 40 |
|  | D | 23 | 25 | 31 | 37 |
| Total consumption (10^4^ tce) | A | 513 | 636 | 868 | 1038 |
|  | B | 367 | 459 | 609 | 732 |
|  | C | 488 | 572 | 683 | 769 |
|  | D | 369 | 426 | 500 | 546 |
|  | City type | S2 | | | |
|  |  | 2015 | 2020 | 2025 | 2030 |
| Share of electricity and renewable energy (%) | A | 43 | 48 | 58 | 64 |
|  | B | 41 | 45 | 54 | 60 |
|  | C | 26 | 30 | 37 | 45 |
|  | D | 23 | 27 | 33 | 41 |
| Total consumption (10^4^ tce) | A | The same as that of S1 | | | |
|  | B |  |  |  |  |
|  | C |  |  |  |  |
|  | D |  |  |  |  |
|  | City type | S3 | | | |
|  |  | 2015 | 2020 | 2025 | 2030 |
| Share of electricity and renewable energy (%) | A | The same as that of S2 | | | |
|  | B |  |  |  |  |
|  | C |  |  |  |  |
|  | D |  |  |  |  |
| Total consumption (10^4^ tce) | A | 513 | 573 | 738 | 831 |
|  | B | 367 | 427 | 535 | 605 |
|  | C | 488 | 544 | 613 | 649 |
|  | D | 369 | 413 | 458 | 471 |
| Cities included | A | Wuhan | | | |
|  | B | Yichang, Xiangyang, Ezhou | | | |
|  | C | Huangshi, Shiyan, Jingmen,Xiaogan,Xianning, Enshi, Qianjiang | | | |
|  | D | Jingzhou, Huanggang, Suizhou, Xiantao, Tianmen, Shennongjia | | | |
